# Exposure to Famine at a Young Age and Unhealthy Lifestyle Behavior Later in Life

**DOI:** 10.1371/journal.pone.0156609

**Published:** 2016-05-31

**Authors:** Heidi P. Fransen, Petra H. M. Peeters, Joline W. J. Beulens, Jolanda M. A. Boer, G. Ardine de Wit, N. Charlotte Onland-Moret, Yvonne T. van der Schouw, H. Bas Bueno-de-Mesquita, Jeljer Hoekstra, Sjoerd G. Elias, Anne M. May

**Affiliations:** 1 Julius Center for Health Sciences and Primary Care, University Medical Center Utrecht, Utrecht, the Netherlands; 2 National Institute for Public Health and the Environment (RIVM), Bilthoven, the Netherlands; 3 School of Public Health, Imperial College London, London, United Kingdom; 4 Department of Gastroenterology and Hepatology, University Medical Center Utrecht, Utrecht, the Netherlands; 5 Dt. of Social & Preventive Medicine, Faculty of Medicine, University of Malaya, Kuala Lumpur, Malaysia; Sookmyung Women's University, REPUBLIC OF KOREA

## Abstract

**Background:**

A healthy diet is important for normal growth and development. Exposure to undernutrition during important developmental periods such as childhood and adolescence can have effects later in life. Inhabitants of the west of the Netherlands were exposed to severe undernutrition during the famine in the last winter of the second World War (1944–1945).

**Objective:**

We investigated if exposure of women to the Dutch famine during childhood and adolescence was associated with an unhealthy lifestyle later in life.

**Design:**

We studied 7,525 women from the Prospect-EPIC cohort, recruited in 1993–97 and aged 0–18 years during the Dutch famine. An individual famine score was calculated based on self-reported information about experience of hunger and weight loss. We investigated the association between famine exposure in early life and four lifestyle factors in adulthood: smoking, alcohol consumption, physical activity level and a Mediterranean-style diet.

**Results:**

Of the 7,525 included women, 46% were unexposed, 38% moderately exposed and 16% severely exposed to the Dutch famine. Moderately and severely exposed women were more often former or current smokers compared to women that did not suffer from the famine: adjusted prevalence ratio 1.10 (95% CI: 1.05; 1.14) and 1.18 (1.12; 1.25), respectively. They also smoked more pack years than unexposed women. Severely exposed women were more often physically inactive than unexposed women, adjusted prevalence ratio 1.32 (1.06; 1.64). Results did not differ between exposure age categories (0–9 and 10–17 years). We found no associations of famine exposure with alcohol consumption and no dose-dependent relations with diet.

**Conclusions:**

Exposure to famine early in female life may be associated with higher prevalence of smoking and physical inactivity later in life, but not with unhealthy diet and alcohol consumption.

## Introduction

A healthy diet is important for normal growth and development, especially during important developmental periods such as childhood and adolescence [1]. The developmental origins of health and disease hypothesis posits that undernutrition during fetal and infant life results in early adaptations of the body, which may lead to chronic disease later in life [2]. This hypothesis is supported by results from Dutch famine studies [3–6].

The Dutch famine took place in the winter of 1944–1945. Inhabitants of the Western part of the Netherlands were exposed to severe undernutrition in the last 6 months of the Second World War. This historical event created a unique opportunity to gain insight into the long-term effects of a relatively short period of transient undernutrition. Because of the short exposure period, it is possible to pinpoint effects to specific growth periods in human life.

Increased risks of overweight, diabetes, coronary heart disease, COPD and asthma have been reported in individuals who were exposed to the Dutch famine [3–6]. Furthermore, famine exposure was associated with an increased risk of breast cancer in one study [7], while others found no clear effects [8]. No associations were found with non-breast cancer risk [9]. The associations between famine exposure early in life and various biological outcomes may be due to biological effects, i.e. epigenetic [10] or hormonal changes [11], or to behavioral reactions following the exposure. The association between undernutrition early in life and different health behaviors later in life has not been investigated in depth before. To the best of our knowledge only one working paper describes the association between undernutrition and dietary intake. Kesternich *et al*. suggested that early-life shocks affect nutritional behavior later in life [12]. Exposure to hunger during childhood was related to an increased fraction of income that was spent on food later in life. However, true food intake was not measured and it was therefore not known if they consumed healthy or unhealthy products. No studies on other lifestyle factors are available. Studies have related adverse childhood experiences and stress during childhood to chronic disease risk later in life [13–15]. Miller *et al* present a model to explain how childhood stress mechanistically leads to higher susceptibility to chronic diseases later in life. Stress during childhood may among others impair self-regulation, resulting in unhealthy lifestyle choices [13]. We hypothesize that exposure to famine early in life is associated to an unhealthy lifestyle later in life. Unhealthy behaviors, such as smoking, drinking, being physically inactive, and eating an unhealthy diet, are important risk factors for many non-communicable diseases [16, 17] and may act as an intermediate factor between famine exposure and chronic disease occurrence later in life. In the present study we therefore investigate if exposure to the Dutch famine during childhood and adolescence is associated with an unhealthy lifestyle later in life. We focus on the lifestyle factors smoking, alcohol consumption, physical activity level and usual diet.

## Materials and Methods

### The Dutch famine

During the Second World War, from October 1944 till April 1945, inhabitants of the occupied Western part of the Netherlands were exposed to famine. Their daily food rations dropped to less than 25% of the pre-famine rations and varied between 400–800 kcal/day [18]. After approximately 6 months of hunger the famine ended abruptly by liberation of the Netherlands in May 1945, and food became available again through supplies of the allied forces. This short period of extreme hunger allows the study of long-term effects of famine exposure.

### The Prospect-EPIC cohort

We investigated the association between famine exposure and an unhealthy lifestyle in the Prospect-EPIC cohort. This is one of two Dutch cohorts of the European Prospective Investigation into Cancer and Nutrition [19, 20]. Between 1993 and 1997 17,357 women were recruited in the Prospect-EPIC cohort. They all participated in the nationwide breast cancer screening program and were living in the city of Utrecht or surroundings. At recruitment, the women completed a general questionnaire (containing among others three questions about exposure to the 1944–1945 famine) and a validated food frequency questionnaire [21, 22], and underwent a physical examination. All participants provided written informed consent before study inclusion. The Prospect-EPIC study complies with the Declaration of Helsinki and was approved by the Institutional Review Board of the University Medical Center Utrecht.

### Exclusion criteria

We excluded participants who answered ‘not applicable’ or ‘I don’t know’ to one or more of the three famine exposure questions (n = 4975). Furthermore, we excluded women who were born after the Dutch famine (n = 2559) or who were >18 years during the famine (N = 481), or who lived outside the Netherlands during the famine (n = 1732), or who had no dietary information available (n = 85). Our final study population consisted of 7,525 women.

### Individual famine score

Participants were asked about their experience of hunger and weight loss during the famine [3]. The questions each contained the answer categories ‘hardly’, ‘little’, and ‘very much’. These categories were combined into a three-point famine exposure score, as previously reported: 1) severely exposed: women who reported being ‘very much’ exposed to both hunger and weight loss; 2) unexposed: women who reported ‘hardly’ being exposed to both hunger and weight loss; and 3) moderately exposed: all others [3].

### Exposure age categories

We divided women into two age categories, using age at start of the famine (October 1^st^, 1944), because we wanted to investigate the effect of famine exposure during different growth periods. These categories were made according to the human life cycle as defined by Bogin [23] and have been used in the Prospect-EPIC cohort before [4]: 0–9 years (childhood, n = 4385), and 10–17 years (adolescence, n = 3140).

### Unhealthy lifestyle factors

#### Smoking

Information on smoking status and smoking intensity was available from the general questionnaire at recruitment (1993–7). Smoking status was defined as current, former or never smoker (categorical). Pack years of smoking (continuous) were calculated as packs (25 cigarettes) smoked per day multiplied by years of smoking. Pack years were available and analyzed for current and former smokers.

#### Alcohol consumption

Information on alcohol consumption from the baseline questionnaire (being a never, former or current drinker) was combined with alcohol intake from the food frequency questionnaire (in grams ethanol per day) and categorized into never drinkers (abstainers), light current drinkers (>0–5 g/day), moderate current drinkers (5–15 g/day) and heavy current drinkers (≥15 g/day). Furthermore, the amount of alcohol intake was analyzed among women who drank ≥1 g/day. For women who drank less, their intake may come from other products than alcoholic drinks, i.e. chocolate candy or sauces.

#### Physical activity level

Physical activity level was assessed in the general questionnaire and categorized according to the validated Cambridge Physical Activity Index into inactive, moderately inactive, moderately active or active [24].

#### Diet

The modified Mediterranean Diet Score (mMDS) was used as a measure of a healthy diet [25]. Compared with the original Mediterranean Diet Score fish and poly-unsaturated fatty acids were additionally included in this score [26]. A high score is associated with lower risk of chronic diseases [27] and in the total EPIC-NL cohort with a longer healthy life expectancy [28]. Information of the food frequency questionnaire was used to score intake of eight components of the mMDS: vegetables; legumes; fruit, nuts and seeds; cereals; fish; the ratio of unsaturated to saturated fatty acids; meat; and dairy products. For the first 6 components intake equal to or above the study population median was assigned a value of 1, and intake below the median a value of 0. For meat and dairy products intake equal to or below the median was assigned a value of 1. Points were summed into the modified Mediterranean Diet Score, ranging from zero to eight points We did not include alcohol consumption in the score, as alcohol consumption was investigated as a separate lifestyle factor. A low self-reported modified Mediterranean Diet Score, i.e. a score below 4, was defined as an unhealthy diet. Furthermore, the score was analyzed continuously.

### Covariates

We used age at start of the famine (1st October 1944) and educational level, which is considered to be a proxy for socioeconomic status, as covariates in our analyses. We categorized levels of education into very low (only primary school), low (lower vocational education), middle (secondary school or intermediate vocational training) and high education (higher vocational training or university). Next, body mass index (BMI) and energy intake (kcal/day) were included as covariates. BMI (kg/m^2^) was calculated from measured weight and height and used as a continuous variable. Energy intake was calculated in kcal/day using food frequency questionnaire data; and used as a continuous variable. For smoking as a covariate, smoking status and intensity were combined and categorized into 8 categories, i.e. current smoker (<15 cigarettes/day, 15–25 cigarettes/day, >25 cigarettes a day, pipe of cigar smoker), former smoker (quit <10 year ago, quit 10–20 year ago, quit >20 year ago) and never smoker.

### Statistical analysis

Missing data on BMI (N = 10) and educational level (N = 9) were imputed, using single imputation regression modelling (SPSS-MVA). Characteristics of the study population are presented according to level of famine exposure as mean and standard deviation or as a percentage. Associations between famine exposure and lifestyle were determined for the total study population and by age category. For categorical variables, we used a Poisson regression model, because an odds ratio will overestimate the effect size because of the high prevalences [29]. Prevalence ratios were estimated for smoking (ever smokers [current or former smokers], vs. never smokers), drinking (heavy alcohol consumption, ≥15 g/day, vs. drinking less than 15 g/day), having an unhealthy diet (mMDS below 4 vs. mMDS of 4 or higher) and physical inactivity (inactive vs the rest, the latter includes participants that were active, moderately active and moderately inactive). The associations between famine exposure and continuous variables, i.e. pack years of smoking (only for current and former smokers), alcohol intake (in grams ethanol/day; only for current drinkers that drink >1 g/day), and modified Mediterranean Diet Score were estimated by linear regression.

Crude and multivariable models are presented. Multivariable models are adjusted for 1) age at start of the famine (October 1^st^, 1944; continuous) and educational level (categorical); 2) all variables in model 1 and BMI, energy intake, physical activity level, alcohol/smoking status and intensity, and mMDS. Covariates were excluded in the analyses where they are the outcome. A P value for linear trend was computed by including the categorical famine exposure score as a continuous variable in the model. We studied whether results were comparable for the two exposure age categories (0–9 and 10–17 years) by including interaction terms in the model. All statistical analyses were conducted using SAS 9.2 (SAS Institute, Cary, US). P-values <0.05 were considered to be statistically significant.

## Results

Characteristics of the study population according to self-reported level of famine exposure are presented in **Table 1**. Of the 7,525 included women, 46% were unexposed, 38% moderately exposed and 16% severely exposed to famine. Participants who were severely exposed to famine were older at start of the famine and more often lower educated.

**Table 1:** Characteristics of the study population at recruitment, according to level of famine exposure, n = 7,525.

			Level of famine exposure		
			Unexposed	Moderately exposed	Severely exposed
Participants	N (%)		3450 (46%)	2838 (38%)	1237 (16%)
Age at start of famine (Oct 1^st^, 1944), in years	Mean (SD)		8.0 (5.3)	8.8 (5.4)	9.1 (5.1)
Aged 0–9 years during famine (childhood)	N (%)		2122 (62%)	1601 (56%)	662 (54%)
Aged 10–18 years during famine (adolescent)	N(%)		1328 (38%)	1237 (44%)	575 (46%)
Age at recruitment (1993–1997), in years	Mean (SD)		58.8 (5.4)	59.5 (5.5)	59.7 (5.2)
BMI, kg/m2	Mean (SD)		26.0 (3.9)	26.2 (4.0)	26.2 (4.2)
Waist, cm	Mean (SD)		83.5 (9.8)	84.4 (9.9)	84.7 (10.4)
Level of education	N (%)	Very low	820 (24%)	627 (22%)	320 (26%)
		Low	1691 (49%)	1355 (48%)	617 (50%)
		Middle	461 (13%)	402 (14%)	152 (12%)
		High	478 (14%)	454 (16%)	148 (12%)
Smoking status	N (%)	Never	1667 (48%)	1246 (44%)	503 (41%)
		Former	1121 (32%)	997 (35%)	457 (37%)
		Current	662 (19%)	595 (21%)	277 (22%)
Physical activity	N (%)	Inactive	206 (6%)	218 (8%)	112 (9%)
level		Moderately inactive	951 (28%)	742 (26%)	288 (23%)
		Moderately active	875 (25%)	728 (26%)	339 (27%)
		Active	1418 (41%)	1150 (41%)	498 (40%)
Alcohol	N (%)	Never	15 (0%)	17 (0%)	12 (1%)
consumption		Light (0–5 g/day)	1917 (56%)	1562 (55%)	749 (61%)
		Moderate (5–15 g/day)	790 (23%)	682 (24%)	228 (18%)
		Heavy (≥ 15 g/day)	728 (21%)	577 (20%)	248 (20%)
Energy intake in kcal/day	Mean (SD)		1800 (420)	1790 (411)	1756 (428)
mMDS, excluding alcohol	Mean (SD)		4.0 (1.5)	4.1 (1.5)	4.0 (1.5)
Unhealthy diet (mMDS<4)	N (%)		1300 (38%)	979 (35%)	474 (38%)

Associations between famine exposure and unhealthy lifestyle factors are presented for the total study population in Tables 2 and 3. Results stratified by age category (0–9 years or 10–17 years at start of the famine) are presented in S1–S7 Tables.

**Table 2:** Categorical analysis: prevalence ratios and 95% CI for smoking, drinking, an unhealthy diet, and physical inactivity, according to level of famine exposure.

Famine exposure level	Crude model	P for trend	Multivariable model 1^1^	P for trend	Multivariable model 2^1^	P for trend	Interaction with age
**Smoking**^2^							
Unexposed	Reference	<0.0001	Reference	<0.0001	Reference	<0.0001	0.23
Moderately	1.09 (1.04; 1.14)		1.09 (1.04; 1.14)		1.10 (1.05; 1.14)		
Severely	1.15 (1.09; 1.21)		1.17 (1.11; 1.24)		1.18 (1.12; 1.25)		
**Drinking**^3^							
Unexposed	Reference	0.37	Reference	0.88	Reference	0.24	0.04
Moderately	0.96 (0.87; 1.06)		0.97 (0.88; 1.06)		0.94 (0.85; 1.03)		
Severely	0.95 (0.84; 1.08)		1.01 (0.89; 1.14)		0.95 (0.84; 1.07)		
**Unhealthy diet**^4^							
Unexposed	Reference	0.60	Reference	0.55	Reference	0.26	0.51
Moderately	0.92 (0.86; 0.98)		0.92 (0.87; 0.99)		0.92 (0.86; 0.98)		
Severely	1.02 (0.94; 1.10)		1.01 (0.93; 1.09)		0.98 (0.91; 1.07)		
**Physical inactivity**^5^							
Unexposed	Reference	<0.0001	Reference	0.0008	Reference	0.081	0.32
Moderately	1.29 (1.07; 1.55)		1.23 (1.03; 1.48)		1.18 (0.99; 1.42)		
Severely	1.52 (1.22; 1.89)		1.42 (1.15; 1.77)		1.32 (1.06; 1.64)		

^1^ multivariable model 1: adjusted for age at start of the famine (October 1, 1944) and educational level, multivariable model 2: adjusted for age at start of the famine, educational level model, BMI, energy intake, physical activity level, smoking status and intensity, alcohol consumption, and mMDS (covariates are excluded if they are the outcome).

^2^ being a former or current smoker

^3^ heavy drinking, ≥15 g/day

^4^ unhealthy diet is defined as mMDS<4 (excluding alcohol)

^5^ being physically inactive; mMDS: modified Mediterranean Diet Score.

**Table 3:** Continuous analysis of the association between famine exposure and pack years of smoking, alcohol consumption, and diet (regression coefficients and 95% CI).

Famine exposure level	N		Crude model	P for trend	Multivariable model 1^1^	P for trend	Multivariable model 2 ^1^	P for trend	Interaction with age
**Pack years of smoking** ^2^		Packyears, mean (SD)							
Unexposed	1684	14.2 (12.9)	Reference	<0.0001	Reference	<0.0001	Reference	<0.0001	0.51
Moderately	1514	15.2 (13.6)	1.01 (0.08; 1.94)		0.95 (0.03; 1.87)		0.98 (0.10; 1.87)		
Severely	696	17.3 (14.1)	3.10 (1.92; 4.29)		2.58 (1.41; 3.75)		2.53 (1.39; 3.66)		
**Alcohol intake**^3^		Ethanol, g/day mean (SD)							
Unexposed	2360	12.8 (13.0)	Reference	0.78	Reference	0.87	Reference	0.36	0.50
Moderately	1949	12.7 (12.7)	-0.15 (-0.93; 0.64)		-0.16 (-0.93; 0.62)		-0.41 (-1.15; 0.33)		
Severely	783	12.7 (14.1)	-0.09 (-1.15; 0.96)		0.20 (-0.85; 1.25)		-0.32 (-1.32; 0.67)		
**Diet** ^4^		mMDS, mean (SD)							
Unexposed	3450	4.0 (1.5)	Reference	0.33	Reference	0.31	Reference	0.10	0.77
Moderately	2838	4.1 (1.5)	0.10 (0.03; 0.17)		0.08 (0.01; 0.16)		0.09 (0.02; 0.17)		
Severely	1237	4.0 (1.5)	0.00 (-0.09; 0.10)		0.02 (-0.08; 0.11)		0.05 (-0.05; 0.14)		

^1^ multivariable model 1: adjusted for age at start of the famine (October 1, 1944) and educational level;multivariable model 2: adjusted for age at start of the famine, educational level model, BMI, energy intake, physical activity level, smoking status and intensity, alcohol consumption, and mMDS (covariates are excluded if they are the outcome)

^2^ includes former and current smokers only

^3^ only current drinkers that drink >1 g/day

^4^ modified Mediterranean Diet Score excluding alcohol; mMDS: modified Mediterranean Diet Score.

Famine exposure was dose-dependently associated with prevalence of ever smoking; adjusted prevalence ratios were 1.10 (95% CI: 1.05; 1.14) and 1.18 (1.12; 1.25) for moderately exposed and severely exposed women, respectively (P for trend <0.0001) **(Table 2**). This was observed in both age categories (**S1 Table**). Famine exposure was also dose-dependently associated with pack years of smoking; moderately exposed women reported smoking 0.98 (0.10; 1.87) more pack years than unexposed women, while severely exposed women reported 2.53 (1.39; 3.66) more pack years (P for trend <0.0001) (**Table 3**). Results were similar across age categories (**S2 Table**), with no significant interaction of famine exposure with age category (P = 0.51).

No association was found between famine exposure and heavy drinking: adjusted prevalence ratios for moderately and severely exposed women compared to unexposed women were 0.94 (0.85; 1.03) and 0.95 (0.84; 1.07), respectively (Table 2). Although a significant interaction with age was found (P = 0.04), in both age categories prevalence ratios were not statistically significant. Famine exposure was not associated with alcohol intake in grams per day, neither in the total population (Table 3), nor in the two age categories (**S4 Table**).

Moderately exposed women less often reported having an unhealthy diet than unexposed women: adjusted prevalence ratio 0.92 (0.86; 0.98) (Table 2). No differences were found between severely exposed and unexposed women. No significant interaction with age was observed (P = 0.51) (**S5 Table**). We also investigated the mMDS continuously. In the total population, moderately exposed women had a 0.08 point (95% CI: 0.00; 0.16) higher mMDS, compared to unexposed women (Table 3). No differences were found between severely exposed and unexposed women, and no interaction with age was found (P = 0.77) (**S6 Table**).

Famine exposure was associated with physical inactivity. Both moderately exposed and severely exposed women were more often physically inactive than unexposed women, adjusted prevalence ratio 1.18 (0.99; 1.42) and 1.32 (1.06; 1.64), respectively (P for trend = 0.08) (Table 2). The dose-dependent relation was more pronounced in the older age category (P for trend = 0.001) (**S7 Table**).

## Discussion

In our study, women who reported severe exposure to famine during their youth were more often smokers and smoked more later in life compared to women who were not exposed. Exposed women were also more often physically inactive. Associations were dose-dependent: stronger exposure to famine was associated with higher prevalence of smoking and physical inactivity. No interactions with age were found. We found no associations of famine exposure with alcohol consumption and no dose-dependent relations with diet.

These results are in accordance with our hypothesis that famine exposure during important developmental periods, such as childhood and adolescence, may relate to an unhealthier lifestyle later in life. However, famine exposure was not associated with alcohol consumption later in life and no clear relations with the modified Mediterranean diet Score were found. In our study, moderately exposed women reported a higher modified Mediterranean Diet Score, indicating that these women ate a healthier diet than the unexposed women. We have no clear explanation for these results, which were especially present in the younger age category (0–9 years old during the famine). It has to be noted, however, that the moderately exposed group is a very diverse group. This group also contained women who were little exposed to hunger or weight loss, or very much exposed to either weight loss or hunger.

Relations of exposure to the Dutch famine with occurrence of chronic diseases later in life have been reported previously. Famine exposure was associated with higher rates of overweight, diabetes, coronary heart disease, COPD and asthma [3–6]. These associations were only partly corrected for unhealthy behaviors. Unhealthy behaviors are important risk factors for these diseases [16, 17] and may act alone or in combination as intermediate factors between famine exposure and chronic disease occurrence later in life. Little information on the association between famine exposure and lifestyle later in life is available. Most studies focused on cognition, which is often related with lifestyle, in children following famine exposure [1, 30] and on prenatal [31] instead of postnatal exposure.

We observed a higher prevalence of smoking and physical inactivity in participants that were severely exposed to the Dutch famine. As we are the first to study this association, there are no other studies to compare our results with. We can only speculate about the biological pathways along which famine exposure early in life may be associated with lifestyle later in life. Severe undernutrition during important developmental periods might impair brain development, as rapid brain development takes place during pregnancy and in the first years of life [1]. However, environment (brain development is affected by experience), timing of the exposure, the degree of exposure and the possibility of recovery might also influence long-term effects of famine exposure on brain function [1]. Vucetic *et al*. studied the effect of early life protein restriction (pre- and postnatal) in mice and found behavioral abnormalities that were dopamine-related [32]. Dopamine plays a role in behavioral responses, and a dysfunction of the dopamine system is associated with neurobehavioral disorders, like addiction. This may explain the results found for smoking, but not the fact that heavy alcohol consumption was not related to famine exposure. Apart from a direct effect of the famine, famine-related stress or war-related stress may play a role in the adoption of unhealthy behaviors [3, 12]. Stressful life events have been associated with higher risk of smoking and drinking in adolescents [33]. Several studies relate adverse childhood experiences and stress during childhood to increased chronic disease risk later in life, among others via unhealthy lifestyle behaviors [13–15]. Miller *et al*. suggest that impaired self-regulation after exposure to stress during childhood creates a proclivity for unhealthy behaviors [13].

The Prospect-EPIC cohort provided us with the unique opportunity to study the long-term relations of famine exposure with lifestyle behavior later in life. Strengths of our study are the large study population and the documented famine period. The exposure had a sudden onset and ending, and took place in a previously well-nourished population. In addition, we were able to calculate individual famine scores from our questionnaire. Usually, place of residence is used as a marker of famine exposure in other studies, which is less accurate. It has to be noted, however, that our individual famine scores are based on self-reported data on the experience of weight loss and hunger, and especially women in the youngest age category may depend on information from their family for the recollection of their exposure. However, in our study, participants who were severely exposed to the famine were older than participants that were moderately exposed or unexposed. This complies with the historical fact that young children were relatively protected from hunger during the war and supports the quality of our data [18]. Our study population included women who participated in the Dutch national breast cancer screening program (participation rate around 78%) [34], resulting in possible selective participation of women with an overall healthier lifestyle [35]. Results are also conditional on survival until recruitment into the Prospect-EPIC study (1993–7). It is possible that women with the unhealthiest lifestyles had already died before the study started. This may have biased our results to an underestimation of the associations. Furthermore, as our study cohort only included women, we do not know if results will be similar for men.

In conclusion, this is the first study that investigated the association between a short period of extreme hunger in early life and the presence of unhealthy lifestyle factors later in life. In women, exposure to famine was associated with a higher prevalence of smoking and physical inactivity, while no clear relations were found with diet and alcohol consumption. Our results imply that, next to having direct biological effects that increase chronic disease risk, famine exposure might indirectly relate to chronic disease risk through unhealthy lifestyle factors.

## Supporting Information

S1 Table Prevalence ratios and 95% CI for smoking status, according to level of famine exposure, stratified by age category. (DOCX)

S2 Table Association between famine exposure and pack years of smoking, stratified by age category, regression coefficients and 95% CI, n = 3,894. (DOCX)

S3 Table Prevalence ratios and 95% CI for being a heavy drinker, according to level of famine exposure, stratified by age category. (DOCX)

S4 Table Association between famine exposure and alcohol intake in grams ethanol per day, stratified by age category, regression coefficients and 95% CI, n = 5,092. (DOCX)

S5 Table Prevalence ratios and 95% CI for having an unhealthy diet, according to level of famine exposure, stratified by age category. (DOCX)

S6 Table Association between famine exposure and modified Mediterranean Diet Score (excluding alcohol), stratified by age category, regression coefficients and 95% CI, n = 7,525. (DOCX)

S7 Table Prevalence ratios and 95% CI for being physically inactive, according to level of famine exposure, stratified by age category. (DOCX)
