# Supplementary material for: Exposure to Famine at a Young Age and Unhealthy Lifestyle Behavior Later in Life
Source: PLoS One. 2016 May 31;11(5):e0156609. doi: 10.1371/journal.pone.0156609 (PMC4887008; doi:10.1371/journal.pone.0156609)
Supplement: S1 Table — (DOCX) [file pone.0156609.s001.docx]

**S1 Table** Prevalence ratios and 95% CI for smoking status ^1^, according to level of famine exposure, stratified by age category.

| **Age category and famine exposure level** | Crude model | P for trend | Multivariable model 1 ^2^ | P for trend | Multivariable  model 2 ^2^ | P for trend |
| --- | --- | --- | --- | --- | --- | --- |
|  |  |  |  |  |  |  |
| **All ages** |  |  |  |  |  |  |
| Unexposed | Reference | <0.0001 | Reference | <0.0001 | Reference | <0.0001 |
| Moderately | 1.09 (1.04; 1.14) |  | 1.09 (1.04; 1.14) |  | 1.10 (1.05; 1.14) |  |
| Severely | 1.15 (1.09; 1.21) |  | 1.17 (1.11; 1.24) |  | 1.18 (1.12; 1.25) |  |
|  |  |  |  |  |  |  |
| **0-9 years** |  |  |  |  |  |  |
| Unexposed | Reference | 0.0040 | Reference | 0.0013 | Reference | 0.0002 |
| Moderately | 1.06 (1.00; 1.12) |  | 1.07 (1.01; 1.13) |  | 1.07 (1.01; 1.13) |  |
| Severely | 1.10 (1.03; 1.18) |  | 1.12 (1.04; 1.20) |  | 1.13 (1.06; 1.21) |  |
|  |  |  |  |  |  |  |
| **10-17 years** |  |  |  |  |  |  |
| Unexposed | Reference | <0.0001 | Reference | <0.0001 | Reference | <0.0001 |
| Moderately | 1.15 (1.06; 1.25) |  | 1.14 (1.06; 1.24) |  | 1.14 (1.06; 1.24) |  |
| Severely | 1.26 (1.15; 1.38) |  | 1.24 (1.13; 1.36) |  | 1.24 (1.13; 1.35) |  |

^1^ being a former or current smoker;
^2^ multivariable model 1: adjusted for age at start of the famine (October 1, 1944) and educational level;
multivariable model 2: adjusted for age at start of the famine, educational level model, BMI, energy intake, physical activity level, alcohol consumption, and mMDS. mMDS: modified Mediterranean Diet Score.
