# Supplementary material for: Exposure to Famine at a Young Age and Unhealthy Lifestyle Behavior Later in Life
Source: PLoS One. 2016 May 31;11(5):e0156609. doi: 10.1371/journal.pone.0156609 (PMC4887008; doi:10.1371/journal.pone.0156609)
Supplement: S2 Table — (DOCX) [file pone.0156609.s002.docx]

**S2 Table** Association between famine exposure and pack years of smoking ^1^, stratified by age category, regression coefficients and 95% CI, n=3,894

| **Age category and famine exposure level** | N | Packyears, mean (SD) | Crude model | P for trend | Multivariable model 1^2^ | P for trend | Multivariable model 2 ^2^ | P for trend |
| --- | --- | --- | --- | --- | --- | --- | --- | --- |
| **All ages** |  |  |  |  |  |  |  |  |
| Unexposed | 1684 | 14.2 (12.9) | Reference | <0.0001 | Reference | <0.0001 | Reference | <0.0001 |
| Moderately | 1514 | 15.2 (13.6) | 1.01 (0.08; 1.94) |  | 0.95 (0.03; 1.87) |  | 0.98 (0.10; 1.87) |  |
| Severely | 696 | 17.3 (14.1) | 3.10 (1.92; 4.29) |  | 2.58 (1.41; 3.75) |  | 2.53 (1.39; 3.66) |  |
|  |  |  |  |  |  |  |  |  |
| **0-9 years** |  |  |  |  |  |  |  |  |
| Unexposed | 1115 | 13.7 (12.5) | Reference | <0.0001 | Reference | 0.0007 | Reference | 0.0004 |
| Moderately | 903 | 14.4 (12.4) | 0.63 (-0.48; 1.73) |  | 0.64 (-0.44; 1.73) |  | 0.72 (-0.33; 1.76) |  |
| Severely | 386 | 16.9 (13.1) | 3.16 (1.70; 4.61) |  | 2.65 (1.21; 4.09) |  | 2.65 (1.26; 4.04) |  |
|  |  |  |  |  |  |  |  |  |
| **10-17 years** |  |  |  |  |  |  |  |  |
| Unexposed | 569 | 15.2 (13.7) | Reference | <0.0001 | Reference | 0.0076 | Reference | 0.0118 |
| Moderately | 611 | 16.5 (15.1) | 1.35 (-0.32; 3.02) |  | 1.47 (-0.18; 3.12) |  | 1.49 (-0.11; 3.10) |  |
| Severely | 310 | 17.9 (15.3) | 2.69 (0.67; 4.71) |  | 2.62 (0.63; 4.60) |  | 2.37 (0.42; 4.31) |  |

^1^ includes current and former smokers only;
^2^ multivariable model 1: adjusted for age at start of the famine (October 1, 1944) and educational level;
multivariable model 2: adjusted for age at start of the famine, educational level model, BMI, energy intake, physical activity level, alcohol consumption, and mMDS. mMDS: modified Mediterranean Diet Score.
