# Supplementary material for: Exposure to Famine at a Young Age and Unhealthy Lifestyle Behavior Later in Life
Source: PLoS One. 2016 May 31;11(5):e0156609. doi: 10.1371/journal.pone.0156609 (PMC4887008; doi:10.1371/journal.pone.0156609)
Supplement: S3 Table — (DOCX) [file pone.0156609.s003.docx]

**S3 Table** Prevalence ratios and 95% CI for being a heavy drinker ^1^, according to level of famine exposure, stratified by age category.

| **Age category and famine exposure level** | Crude model | P for trend | Multivariable model 1 ^2^ | P for trend | Multivariable model 2 ^2^ | P for trend |
| --- | --- | --- | --- | --- | --- | --- |
| **All ages** |  |  |  |  |  |  |
| Unexposed | Reference | 0.37 | Reference | 0.88 | Reference | 0.24 |
| Moderately | 0.96 (0.87; 1.06) |  | 0.97 (0.88; 1.06) |  | 0.94 (0.85; 1.03) |  |
| Severely | 0.95 (0.84; 1.08) |  | 1.01 (0.89; 1.14) |  | 0.95 (0.84; 1.07) |  |
|  |  |  |  |  |  |  |
| **0-9 years** |  |  |  |  |  |  |
| Unexposed | Reference | 0.81 | Reference | 0.82 | Reference | 0.57 |
| Moderately | 0.98 (0.87; 1.10) |  | 0.97 (0.87; 1.09) |  | 0.94 (0.84; 1.05) |  |
| Severely | 0.99 (0.85; 1.15) |  | 1.04 (0.89; 1.21) |  | 0.98 (0.85; 1.14) |  |
|  |  |  |  |  |  |  |
| **10-17 years** |  |  |  |  |  |  |
| Unexposed | Reference | 0.84 | Reference | 0.56 | Reference | 0.17 |
| Moderately | 1.00 (0.83; 1.19) |  | 0.95 (0.79; 1.13) |  | 0.91 (0.76; 1.08) |  |
| Severely | 0.98 (0.77; 1.23) |  | 0.94 (0.75; 1.19) |  | 0.87 (0.69; 1.08) |  |

^1^ heavy drinking, =>15 g/day ^2^ multivariable model 1: adjusted for age at start of the famine (October 1, 1944) and educational level;
multivariable model 2: adjusted for age at start of the famine, educational level model, BMI, energy intake, physical activity level, smoking status and intensity, and mMDS. mMDS: modified Mediterranean Diet Score.
