# Supplementary material for: Exposure to Famine at a Young Age and Unhealthy Lifestyle Behavior Later in Life
Source: PLoS One. 2016 May 31;11(5):e0156609. doi: 10.1371/journal.pone.0156609 (PMC4887008; doi:10.1371/journal.pone.0156609)
Supplement: S4 Table — (DOCX) [file pone.0156609.s004.docx]

**S4 Table** Association between famine exposure and alcohol intake in grams ethanol per day ^1^, stratified by age category, regression coefficients and 95% CI, n=5,092.

| **Age category and famine exposure level** | N | Mean (SD) | Crude model | P for trend | Multivariable model 1 ^2^ | P for trend | Multivariable model 2 ^2^ | P for trend |
| --- | --- | --- | --- | --- | --- | --- | --- | --- |
| **All ages** |  |  |  |  |  |  |  |  |
| Unexposed | 2360 | 12.8 (13.0) | Reference | 0.78 | Reference | 0.87 | Reference | 0.36 |
| Moderately | 1949 | 12.7 (12.7) | -0.15 (-0.93; 0.64) |  | -0.16 (-0.93; 0.62) |  | -0.41 (-1.15; 0.33) |  |
| Severely | 783 | 12.7 (14.1) | -0.09 (-1.15; 0.96) |  | 0.20 (-0.85; 1.25) |  | -0.32 (-1.32; 0.67) |  |
|  |  |  |  |  |  |  |  |  |
| **0-9 years** |  |  |  |  |  |  |  |  |
| Unexposed | 1552 | 13.8 (13.6) | Reference | 0.78 | Reference | 0.89 | Reference | 0.31 |
| Moderately | 1179 | 13.6 (13.6) | -0.19 (-1.22; 0.85) |  | -0.29 (-1.32; 0.74) |  | -0.51 (-1.49; 0.46) |  |
| Severely | 447 | 13.7 (14.3) | -0.12 (-1.56; 1.31) |  | 0.07 (-1.35; 1.50) |  | -0.53 (-1.88; 0.82) |  |
|  |  |  |  |  |  |  |  |  |
| **10-17 years** |  |  |  |  |  |  |  |  |
| Unexposed | 808 | 10.9 (11.6) | Reference | 0.46 | Reference | 0.61 | Reference | 0.83 |
| Moderately | 770 | 11.2 (11.2) | 0.30 (-0.87; 1.47) |  | 0.06 (-1.11; 1.23) |  | -0.21 (-1.33; 0.90) |  |
| Severely | 336 | 11.5 (13.7) | 0.53 (-0.98; 2.04) |  | 0.44 (-1.06; 1.94) |  | -0.09 (-1.53; 1.35) |  |

^1^ only current drinkers (>1 g/day);
^2^ multivariable model 1: adjusted for age at start of the famine (October 1, 1944) and educational level;
multivariable model 2: adjusted for age at start of the famine, educational level model, BMI, energy intake, physical activity level, smoking status and intensity, and mMDS. mMDS: modified Mediterranean Diet Score.
