# Supplementary material for: Exposure to Famine at a Young Age and Unhealthy Lifestyle Behavior Later in Life
Source: PLoS One. 2016 May 31;11(5):e0156609. doi: 10.1371/journal.pone.0156609 (PMC4887008; doi:10.1371/journal.pone.0156609)
Supplement: S5 Table — (DOCX) [file pone.0156609.s005.docx]

**S5 Table** Prevalence ratios and 95% CI for having an unhealthy diet^1^, according to level of famine exposure, stratified by age category.

| **Age category and famine exposure level** | Crude model | P for trend | Multivariable model 1 ^2^ | P for trend | Multivariable model 2 ^2^ | P for trend |
| --- | --- | --- | --- | --- | --- | --- |
| **All ages** |  |  |  |  |  |  |
| Unexposed | Reference | 0.60 | Reference | 0.55 | Reference | 0.26 |
| Moderately | 0.92 (0.86; 0.98) |  | 0.92 (0.87; 0.99) |  | 0.92 (0.86; 0.98) |  |
| Severely | 1.02 (0.94; 1.10) |  | 1.01 (0.93; 1.09) |  | 0.98 (0.91; 1.07) |  |
|  |  |  |  |  |  |  |
| **0-9 years** |  |  |  |  |  |  |
| Unexposed | Reference | 0.44 | Reference | 0.32 | Reference | 0.18 |
| Moderately | 0.89 (0.82; 0.97) |  | 0.90 (0.82; 0.98) |  | 0.89 (0.82; 0.97) |  |
| Severely | 1.01 (0.90; 1.13) |  | 0.99 (0.89; 1.11) |  | 0.97 (0.87; 1.09) |  |
|  |  |  |  |  |  |  |
| **10-17 years** |  |  |  |  |  |  |
| Unexposed | Reference | 0.98 | Reference | 0.76 | Reference | 0.80 |
| Moderately | 0.94 (0.85; 1.04) |  | 0.96 (0.87; 1.07) |  | 0.95 (0.86; 1.06) |  |
| Severely | 1.02 (0.90; 1.16) |  | 1.04 (0.92; 1.17) |  | 1.00 (0.88; 1.13) |  |

^1^ unhealthy diet is defined as mMDS<4 (excluding alcohol);
^2^ multivariable model 1: adjusted for age at start of the famine (October 1, 1944) and educational level;
multivariable model 2: adjusted for age at start of the famine, educational level model, BMI, energy intake, physical activity level, smoking status and intensity, and alcohol consumption.
