# Supplementary material for: Exposure to Famine at a Young Age and Unhealthy Lifestyle Behavior Later in Life
Source: PLoS One. 2016 May 31;11(5):e0156609. doi: 10.1371/journal.pone.0156609 (PMC4887008; doi:10.1371/journal.pone.0156609)
Supplement: S6 Table — (DOCX) [file pone.0156609.s006.docx]

**S6 Table** Association between famine exposure and modified Mediterranean Diet Score (excluding alcohol), stratified by age category, regression coefficients and 95% CI, n=7,525.

| **Age category and famine exposure level** | N | mMDS, mean (SD) | Crude model | P for trend | Multivariable model 1 ^1^ | P for trend | Multivariable model 2 ^1^ | P for trend |
| --- | --- | --- | --- | --- | --- | --- | --- | --- |
| **All ages** |  |  |  |  |  |  |  |  |
| Unexposed | 3450 | 4.0 (1.5) | Reference | 0.33 | Reference | 0.31 | Reference | 0.10 |
| Moderately | 2838 | 4.1 (1.5) | 0.10 (0.03; 0.17) |  | 0.08 (0.01; 0.16) |  | 0.09 (0.02; 0.17) |  |
| Severely | 1237 | 4.0 (1.5) | 0.00 (-0.09; 0.10) |  | 0.02 (-0.08; 0.11) |  | 0.05 (-0.05; 0.14) |  |
|  |  |  |  |  |  |  |  |  |
| **0-9 years** |  |  |  |  |  |  |  |  |
| Unexposed | 2122 | 4.0 (1.5) | Reference | 0.32 | Reference | 0.18 | Reference | 0.09 |
| Moderately | 1601 | 4.1 (1.5) | 0.13 (0.03; 0.22) |  | 0.12 (0.03; 0.22) |  | 0.13 (0.04; 0.23) |  |
| Severely | 662 | 4.0 (1.6) | 0.00 (-0.13; 0.13) |  | 0.03 (-0.10; 0.16) |  | 0.05 (-0.07; 0.18) |  |
|  |  |  |  |  |  |  |  |  |
| **10-17 years** |  |  |  |  |  |  |  |  |
| Unexposed | 1328 | 4.0 (1.5) | Reference | 0.70 | Reference | 0.97 | Reference | 0.56 |
| Moderately | 1237 | 4.0 (1.4) | 0.06 (-0.05; 0.18) |  | 0.03 (-0.08; 0.14) |  | 0.04 (-0.07; 0.15) |  |
| Severely | 575 | 4.0 (1.5) | 0.01 (-0.14; 0.15) |  | -0.02 (-0.16; 0.13) |  | 0.03 (-0.11; 0.17) |  |

^1^ multivariable model 1: adjusted for age at start of the famine (October 1, 1944) and educational level;
multivariable model 2: adjusted for age at start of the famine, educational level model, BMI, energy intake, physical activity level, smoking status and intensity, and alcohol consumption.
