# Supplementary material for: Exposure to Famine at a Young Age and Unhealthy Lifestyle Behavior Later in Life
Source: PLoS One. 2016 May 31;11(5):e0156609. doi: 10.1371/journal.pone.0156609 (PMC4887008; doi:10.1371/journal.pone.0156609)
Supplement: S7 Table — (DOCX) [file pone.0156609.s007.docx]

**S7 Table** Prevalence ratios and 95% CI for being physically inactive, according to level of famine exposure, stratified by age category.

| **Age category and famine exposure level** | Crude model | P for trend | Multivariable model 1^1^ | P for trend | Multivariable model 2^1^ | P for trend |
| --- | --- | --- | --- | --- | --- | --- |
| **All ages** |  |  |  |  |  |  |
| Unexposed | Reference | <0.0001 | Reference | 0.0008 | Reference | 0.081 |
| Moderately | 1.29 (1.07; 1.55) |  | 1.23 (1.03; 1.48) |  | 1.18 (0.99; 1.42) |  |
| Severely | 1.52 (1.22; 1.89) |  | 1.42 (1.15; 1.77) |  | 1.32 (1.06; 1.64) |  |
|  |  |  |  |  |  |  |
| **0-9 years** |  |  |  |  |  |  |
| Unexposed | Reference | 0.21 | Reference | 0.34 | Reference | 0.48 |
| Moderately | 1.24 (0.93; 1.65) |  | 1.23 (0.92; 1.63) |  | 1.19 (0.90; 1.59) |  |
| Severely | 1.20 (0.82; 1.76) |  | 1.11 (0.76; 1.63) |  | 1.07 (0.73; 1.56) |  |
|  |  |  |  |  |  |  |
| **10-17 years** |  |  |  |  |  |  |
| Unexposed | Reference | 0.0017 | Reference | 0.0008 | Reference | 0.0010 |
| Moderately | 1.24 (0.98; 1.57) |  | 1.25 (0.98; 1.58) |  | 1.26 (0.99; 1.59) |  |
| Severely | 1.57 (1.20; 2.05) |  | 1.63 (1.24; 2.13) |  | 1.60 (1.22; 2.09) |  |

^1^ multivariable model 1: adjusted for age at start of the famine (October 1, 1944) and educational level;
multivariable model 2: adjusted for age at start of the famine, educational level model, BMI, energy intake, smoking status and intensity, alcohol consumption, and mMDS. mMDS: modified Mediterranean Diet Score.
